# Icaritin, a novel FASN inhibitor, exerts anti-melanoma activities through IGF-1R/STAT3 signaling

**DOI:** 10.18632/oncotarget.9984

**Published:** 2016-06-13

**Authors:** Jinfeng Wu, Juan Du, Xiuqiong Fu, Bin Liu, Huihui Cao, Ting Li, Tao Su, Jinhua Xu, Anfernee Kai-Wing Tse, Zhi-Ling Yu

**Affiliations:** ^1^ Center for Cancer and Inflammation Research, School of Chinese Medicine, Hong Kong Baptist University, Kowloon Tong, Hong Kong; ^2^ Department of Dermatology, Huashan Hospital, Fudan University, Shanghai, China; ^3^ School of Traditional Chinese Medicine, Southern Medical University, Guangzhou, China; ^4^ Institute of Integrated Bioinfomedicine and Translational Science, HKBU Shenzhen Research Institute and Continuing Education, Shenzhen, China

**Keywords:** icaritin, melanoma, STAT3, IGF-1R, FASN

## Abstract

Icaritin (IT) is a flavonoid isolated from *Herba Epimedii*. In this study, we evaluated the anti-melanoma activities of IT, and determined its cytotoxic mechanism. We found that IT exerted cytotoxicity to melanoma cells. Furthermore, IT induced melanoma cell apoptosis, which was accompanied with PARP cleavage. Mechanistically, IT suppressed p-STAT3 (tyr705) level in parallel with increases of p-STAT3 (ser727), p-ERK and p-AKT. IT significantly inhibited STAT3 nuclear translocation and reduced the levels of STAT3 -targeted genes. IT also inhibited IGF-1-induced STAT3 activation through down-regulation of total IGF-1R level. No dramatic changes in IGF-1R mRNA levels were observed in IT-treated cells, suggesting that IT acted primarily at a post-transcriptional level. Using molecular docking analysis, IT was identified as a novel fatty acid synthase (FASN) inhibitor. We found that IT reduced the level of total IGF-1R via FASN inhibition. In summary, we reported that IT exerted anti-melanoma activities, and these effects were partially due to inhibition of FASN/IGF-1R/STAT3 signaling.

## INTRODUCTION

Malignant melanoma is the most aggressive form of skin cancer. Although it accounts for less than 2% of all skin cancer cases, it is responsible for 75 percent of deaths from skin cancers [1]. The 5-year survival rate for patients afflicted with metastatic melanoma is approximately 15% [2]. Surgical treatment of early melanoma leads to 90% cure rates, unresectable advanced melanoma is notorious for its intrinsic resistance to chemotherapy, aggressive clinical behavior, and tendency to rapidly metastasize [3].

Targeted therapeutic agents (vemurafenib, trametinib, and imatinib, etc.) show promise in the survival rates in patients with advanced melanoma [4, 5]. However, the majority of patients who respond to the targeted therapies eventually develop resistance and disease progression [6]. Therefore, novel agents need to be developed for overcoming the limitations of the current therapeutic agents.

Signal transducer and activator of transcription 3 (STAT3) is emerging as a therapeutic target in melanoma [7, 8]. In malignant cells, STAT3 functions in regulating cell proliferation, angiogenesis, metastasis and inhibition of apoptosis [9, 10]. Importantly, activation of STAT3 signaling is a negative prognostic factor in human cutaneous melanoma [11]. STAT3 can be activated by cytokines (IL-6, IFN-α), growth factors (EGF, IGF-1 and PDGF, etc) and non-receptor tyrosine kinases (Src and all the JAK family proteins) [12].

Insulin-like growth factor 1 (IGF-1) is one of these growth factors that play important roles in the development and growth of multiple tumors and in the prevention of apoptosis [2]. The biological actions of IGF-1 are mediated through the ligand-induced activation of IGF-1 receptor (IGF-1R), a transmembrane tyrosine kinase linked to mitogen-activated protein kinase (MAPK), phosphatidylinositol-3 kinase/Akt(PI3K/AKT), and STAT3 signal-transduction cascades [13]. IGF1-R overexpression increases tumor progression [14, 15], and decreases of IGF-1R expression or activity causes tumor growth arrest and apoptosis [13, 16]. In addition, IGF-1R disruption increases the sensitivity of melanoma cells to radiotherapy and tumor necrosis factor (TNF)-related apoptosis-inducing ligand (TRAIL)-induced apoptosis [17, 18]. Acquired resistance to BRAF inhibitors can be overcomed by cotargeting MEK and IGF-1R [3].

Phytochemicals, such as flavonoids, represent a source of relatively nontoxic, orally available, and affordable compounds that are known to affect several different cancer-related pathways. Epidemiologic studies have shown a correlation between increased dietary intake of flavonoids and reduced risk of cancer [19]. Icaritin (IT) is one of these flavonoids, which is isolated from *Herba Epimedii* [20]. *Herba Epimedii* is a well-known Chinese herb with proven efficacy in treating cardiovascular diseases, and osteoporosis, and in improving sexual and neurological functions [21]. Recent studies demonstrated that IT might be a novel anticancer agent against a variety of tumor cell lines, including human endometrial carcinoma, hepatic carcinoma, prostate carcinoma and chronic myeloid leukemia cells [22–25].

In this study, we evaluated the anti-melanoma activities of IT, and determined the cytotoxic mechanism through IGF-1R/STAT3 pathway.

## RESULTS

### IT was cytotoxic to melanoma cells *in vitro*

**Figure 1:**
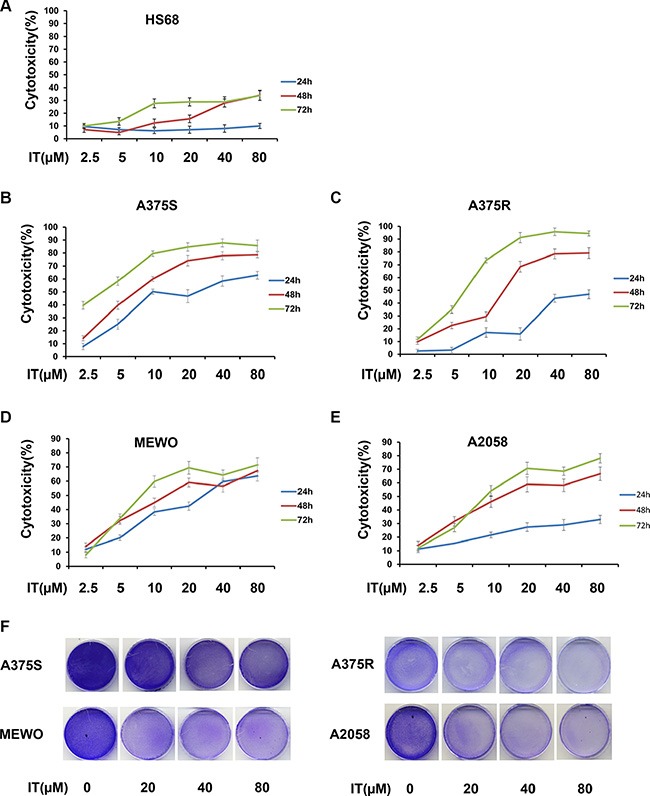
IT was cytotoxic to melanoma cells (**A**) Chemical structure of icaritin (IT). After treatment with various concentrations of IT (2.5, 5, 10, 20, 40, and 80 μM) or vehicle control for 24 h, 48 h or 72 h, the cytotoxicity to HS68 (**B**), A375S (**C**), A375R (**D**), MEWO (**E**), and A2058 (**F**) cells were measured by MTT assay. (**G**) The growth inhibition effects of IT(20, 40, and 80 μM)on melanoma cells were measured by crystal violet assay and photographs were showed after IT treatment for 72 h.

The cytotoxicity of IT was determined by MTT assay (Figure 1A–1E), and crystal violet staining (Figure 1F). IT treatment (2.5, 5, 10, 20, 40 and 80 μM; 24, 48, and 72 h) significantly induced cytotoxicity to human melanoma A375S (parental cells, sensitive to BRAF inhibitor), A375R (resistant to BRAF inhibitor), A2058, and MEWO cells in a time- and dose-dependent manner. The 50% inhibitory concentration (IC50) of IT for 72 h was 2.7, 6.9, 14, and 15.6 μM in A375S, A375R, A2058, and MEWO cells, respectively. IT treatment only showed minor cytotoxicity to human skin fibroblast cells (HS68).

### IT induced melanoma cells apoptosis

**Figure 2:**
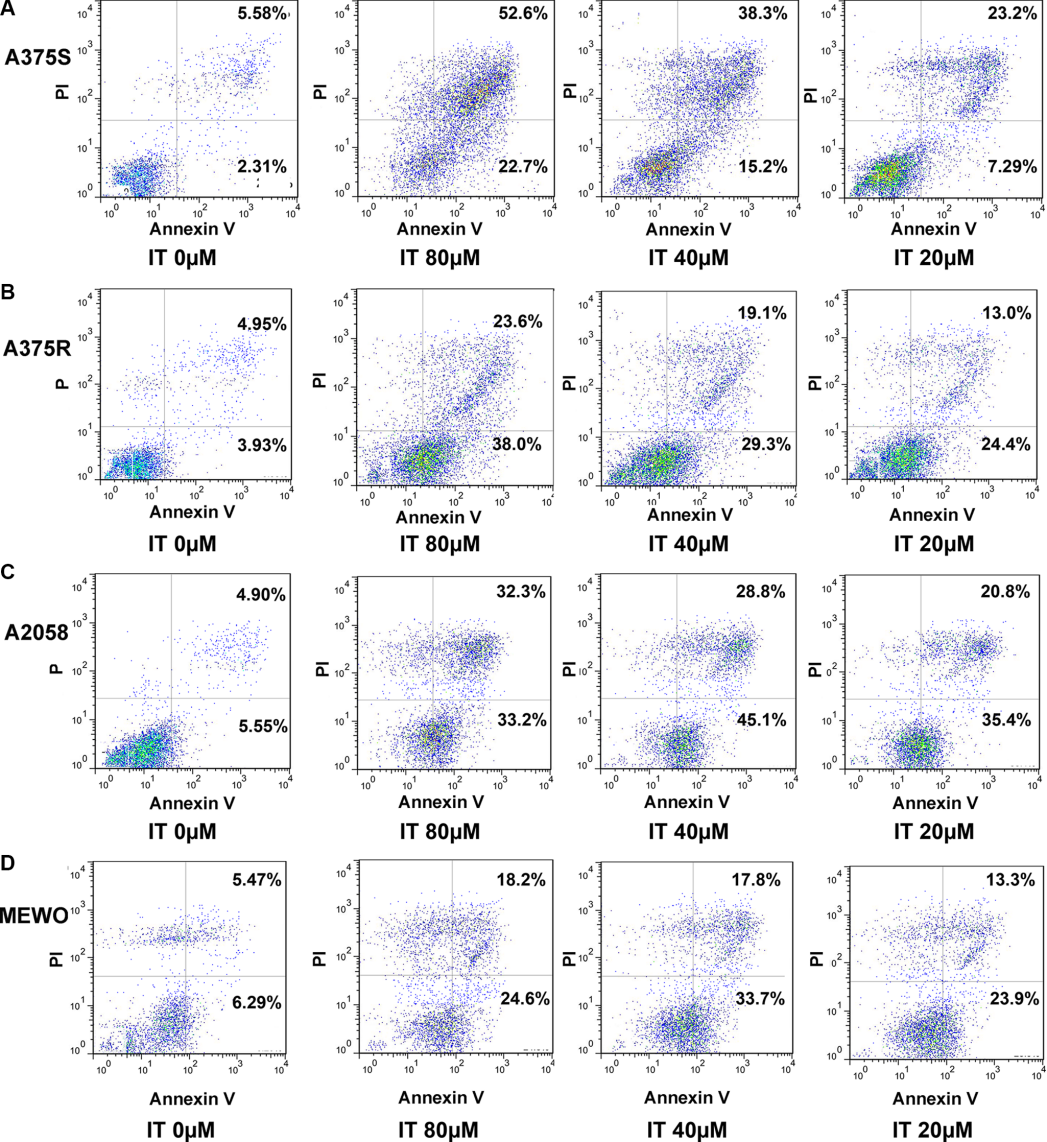
Scatter plot pictures of IT-induced apoptotic melanoma cells The A375S (**A**), A375R (**B**), A2058 (**C**), and MEWO (**D**) cells were treated with various concentrations of IT (20, 40, and 80 μM) or vehicle control for 72 h. Apoptosis was analyzed by flow cytometry using Annexin V/PI double staining. Early apoptotic cells are defined as annexin V+/PI−, whereas late apoptotic/necrotic cells are defined as annexin V+/PI+.

**Figure 3:**
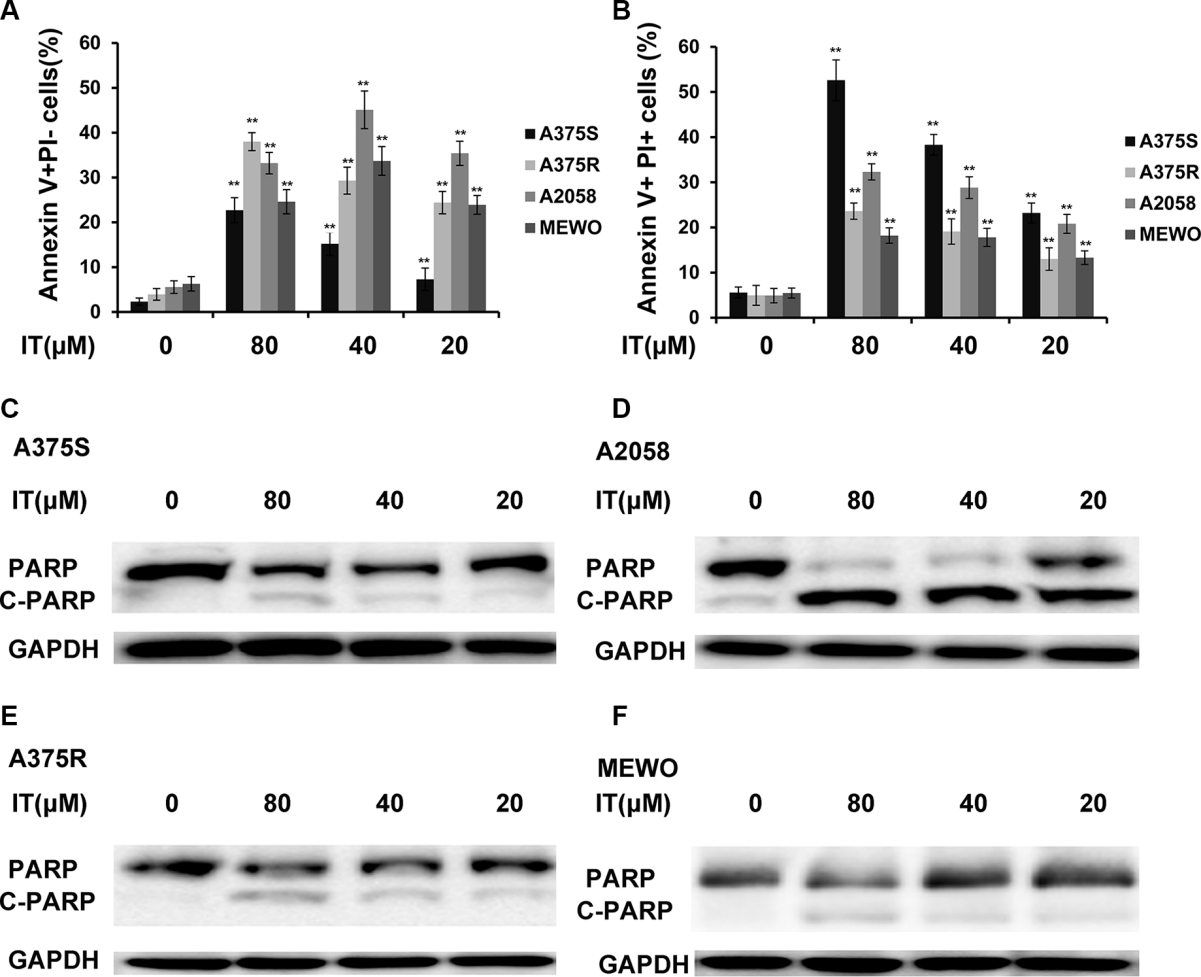
IT induced melanoma cells apoptosis with PARP cleavage Percentages of annexin V+/PI− (**A**) and annexin V+/PI+ (**B**) cells were presented as the mean ± SD of three independent experiments. **indicate *P* < 0.01, as compared with vehicle control group. PARP cleavage in melanoma cells (**C**) A375S; (**D**) A2058; (**E**) A375R; (**F**) MEWO) after treated with various concentrations of IT (20, 40, and 80 μM) or vehicle control for 48 h were detected by western blot analysis.

To determine whether IT would induce apoptosis in melanoma cells, the percentages of Annexin V+/PI- (early apoptotic cells) and Annexin V+ /PI (late apoptotic/necrotic cells) cells were measured by flow cytometry (Figure 2A–2D, and Figure 3A, 3B). IT treatment (20, 40 and 80 μM) for 72 h significantly increased both early and late apoptosis in human melanoma A375S, A375R, A2058, and MEWO cells, as compared with vehicle control group (*P* < 0.01). PARP is one of the terminal pro-apoptotic proteins, and is cleaved to produce the active forms. The effects of IT on PARP and cleaved PARP level (Figure 3C–3F) were determined by western blot analysis. As predicted, IT treatment for 48 h markedly increased the level of cleaved PARP in human melanoma A375S, A375R, A2058, and MEWO cells.

### IT inhibited STAT3 activation and nuclear localization in melanoma cells

**Figure 4:**
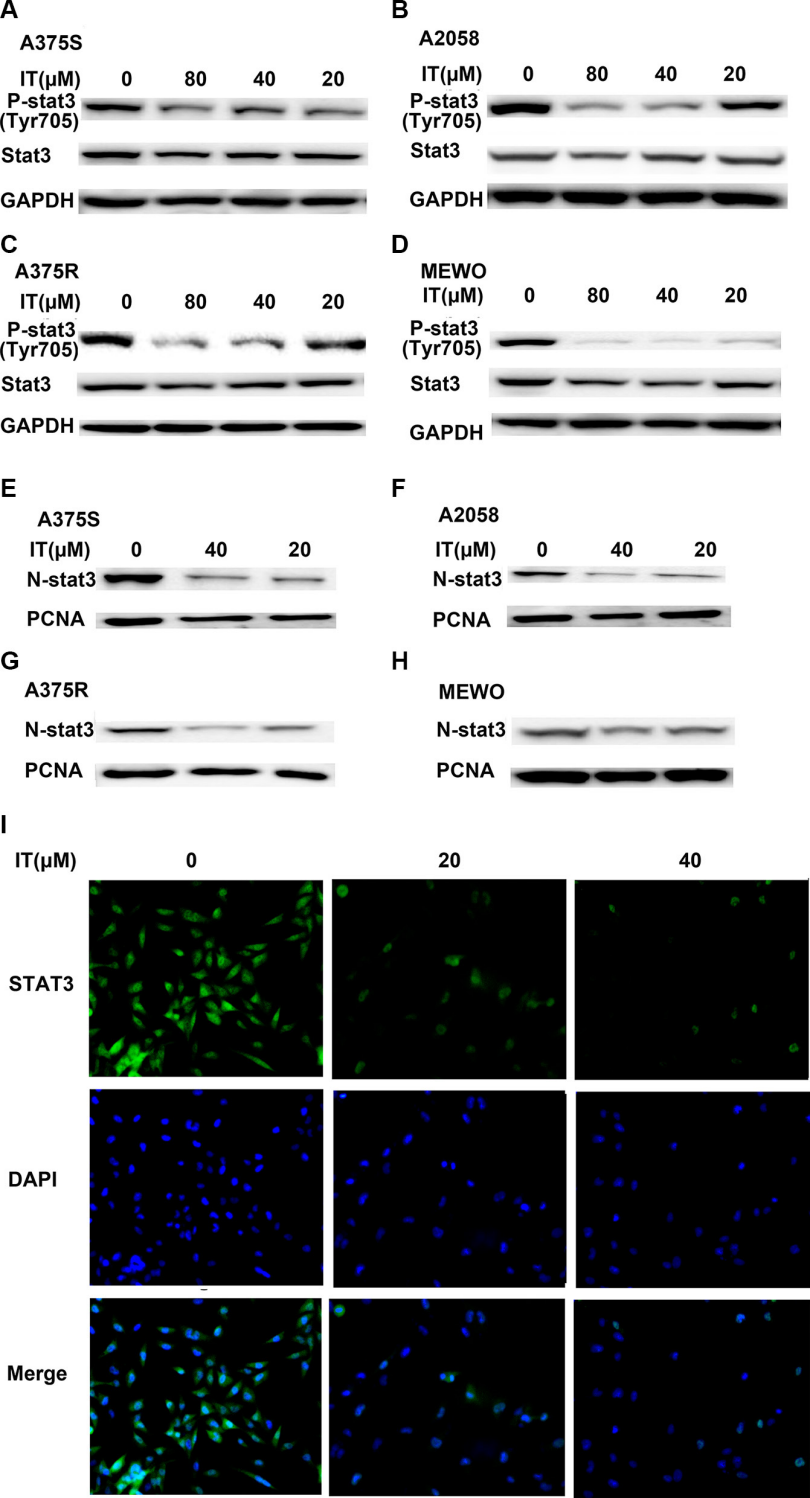
IT inhibited STAT3 activation and nuclear localization in melanoma cells A375S, A2058, A375R, and MEWO cells were treated with various concentrations of IT (20, 40, and 80 μM) or vehicle control for 24 h, and then total cell lysates (**A**, **B**, **C**, and **D**) or nuclear lysates (**E**, **F**, **G**, and **H**) were extracted for western blot analysis by using antibodies specific to p- STAT3 (tyr705) or STAT3. GAPDH or PCNA was used as loading control for total protein or nuclear protein, respectively. For immunostaining analysis (**I**, ×100), A375 cells were treated with IT (0, 20, and 40 μM) for 6 h, the expression of STAT3 was analyzed using a specific mAb and an Alexa Fluor-488-conjugated secondary antibody. The nuclei were stained with DAPI.

It has been well recognized that constitutive phosphorylation/activation of STAT3 contributes to the development and growth of melanoma [26]. Therefore, we investigated whether IT inhibited the activation of STAT3. As shown in Figure 4A–4D, IT treatment (20, 40 and 80 μM) for 24 h decreased the phosphorylated STAT3 at the tyrosine705 (tyr705) site in a dose-dependent manner in human melanoma A375S, A375R, A2058 and MEWO cells. The decreases of total STAT3 were also observed after IT treatment in the four melanoma cell lines. STAT3 dimerization can be induced by phosphorylation at tyr705 site, which then leads to nuclear translocation and DNA binding [6]. Hence, we examined whether IT inhibited the nuclear localization of STAT3. As demonstrated in Figure 4E–4H, the levels of STAT3 in nuclear fractions were markedly reduced by IT treatment (20 and 40 μM) for 24 h. in addition, immunostaining analysis (Figure 4I) showed that both total and nuclear STAT3 protein were decreased by IT treatment (20 and 40 μM) for 6 h.

### IT inhibitedSTAT3 target genes expression in melanoma cells

**Figure 5:**
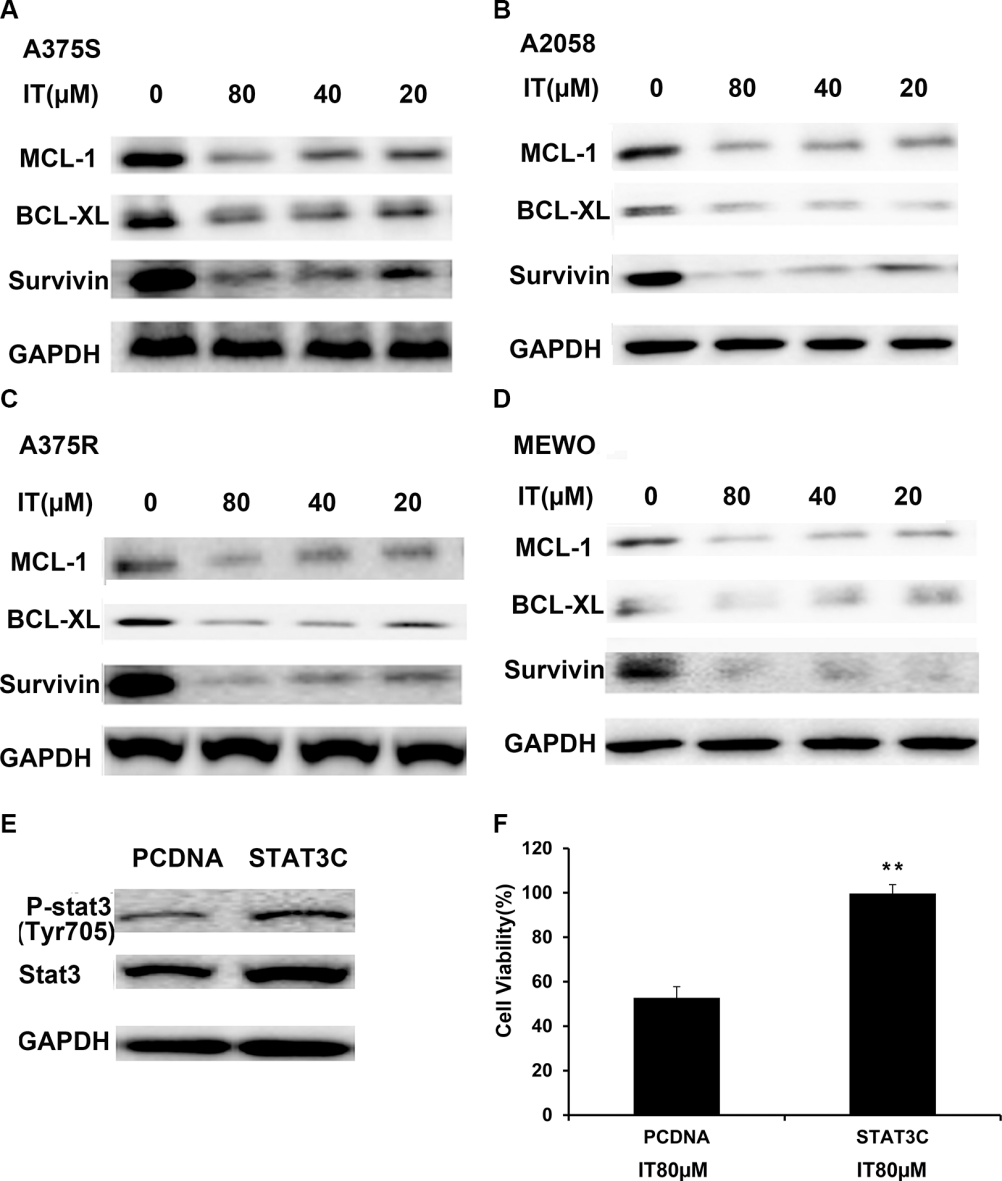
IT inhibited STAT3 target genes expression, while overexpression of STAT3 partially reversed IT-induced growth inhibition A375S (**A**), A2058 (**B**), A375R (**C**), and MEWO (**D**) cells were treated with various concentrations of IT (20, 40, and 80 μM) or vehicle control for 72 h, and then total cell lysates were extracted for western blot analysis using antibodies specific to MCL-1, BCL-XL, and survivin. A375S cells were transiently transfected with STAT3-C or pCDNA for 48 h. (**E**) Western blot analysis of p-STAT3 (tyr705) and STAT3 expression in transfected cells. (**F**) After transfection for 48 h, the cells were treated with IT (80 μM) for 24 h, and then the cell viability was determined by MTT assay. **indicates *P* < 0.01, as compared with vector control.

Survivin, BCL-XL, and MCL-1 have been identified as STAT3- targeted genes, which played important roles in melanoma cell growth and survival [27]. Western blot analysis was employed to determine the effects of IT on STAT3 -targeted genes. As demonstrated in Figure 5A–5D, IT treatment (20, 40 and 80 μM) for 72 h markedly decreased the levels of survivin, BCL-XL, and MCL-1 in human melanoma A375S, A375R, A2058, and MEWO cells.

### Overexpression of STAT3 rescued IT-induced growth inhibition in melanoma cells

To further clarify whether IT-induced melanoma growth inhibition is correlated with STAT3 inactivation, A375Scells were transiently transfected with constitutively active STAT3 plasmid (STAT3-C) for 48 h, followed by IT treatment for another 24 h, and then the cell viability was determined by MTT assay. Western blot analysis (Figure 5E) demonstrated that transfection of STAT3-C in A375 cells resulted in remarkable increases in both STAT3 and p-STAT3 levels, as compared with vector control transfection. After IT 80 μM treatment for 24 h, IT-induced growth inhibition in STAT3-C transfection group was rescued, as compared with that in vector control transfection group (*P* < 0.01, Figure 5F).

### IT activated AKT and ERK signaling in melanoma cells

**Figure 6:**
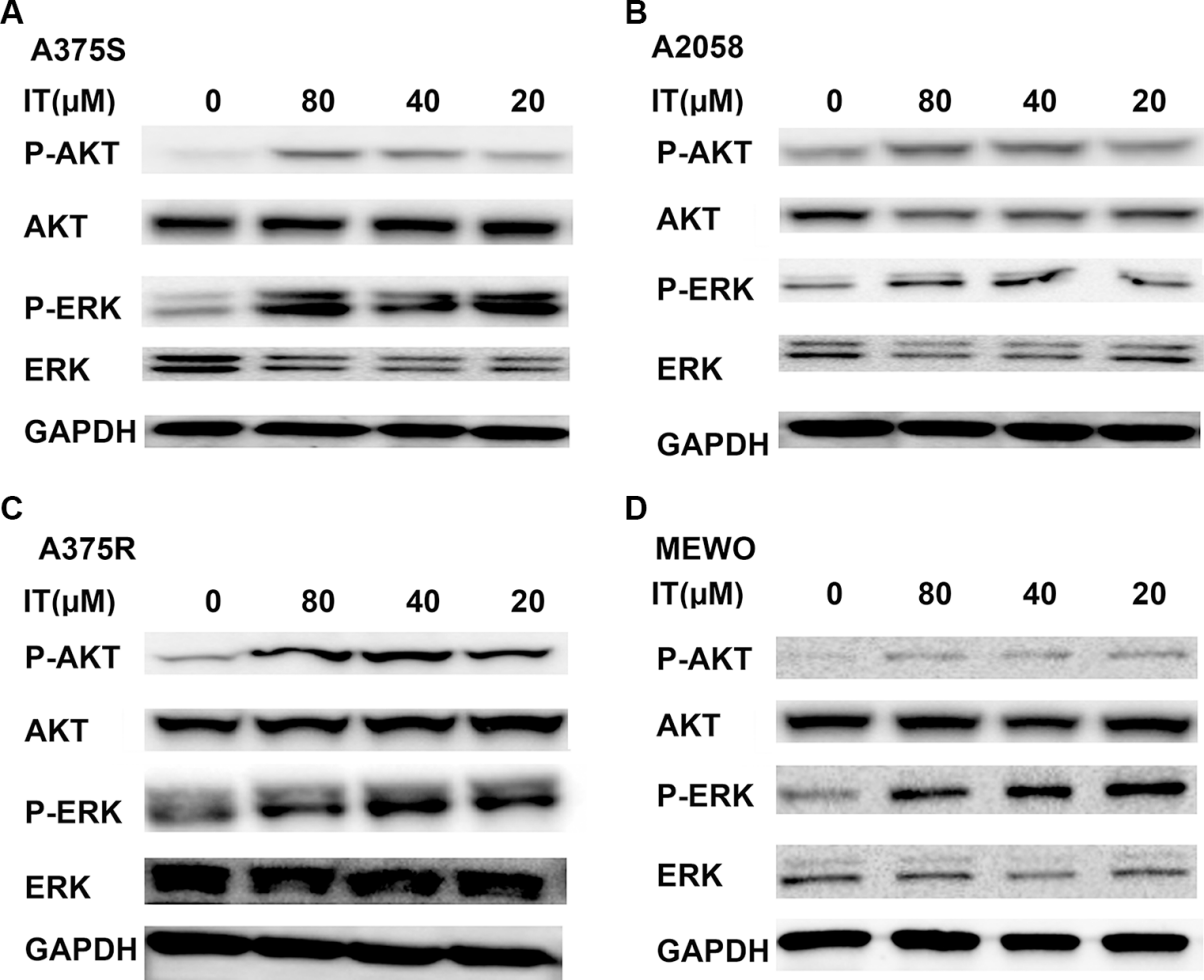
IT activated AKT and ERK signaling in melanoma cells A375S (**A**), A2058 (**B**), A375R (**C**), and MEWO (**D**) cells were treated with various concentrations of IT (20, 40, and 80 μM) or vehicle control for 24 h, and then cell lysates were extracted for western blot analysis using antibodies specific to p-AKT (ser473), AKT, p-ERK (Thr202/Tyr204), and ERK.

It has been reported that the ERK and AKT pathways are two major constitutively activated pro-survival signaling pathways in melanoma [28]. Therefore, we investigated whether IT would inhibit these two signaling pathways. In contrast to the inhibition of STAT3 phosphorylation at tyr705site, IT treatment (20, 40 and 80 μM) for 24 h significantly increased AKT (ser473) and ERK (Thr202/Tyr204) phosphorylation in human melanoma A375S, A375R, A2058, and MEWO cells (Figure 6A–6D).

### Blockade of AKT and ERK activation partially reversed IT-induced STAT3 inhibition in melanoma cells

**Figure 7:**
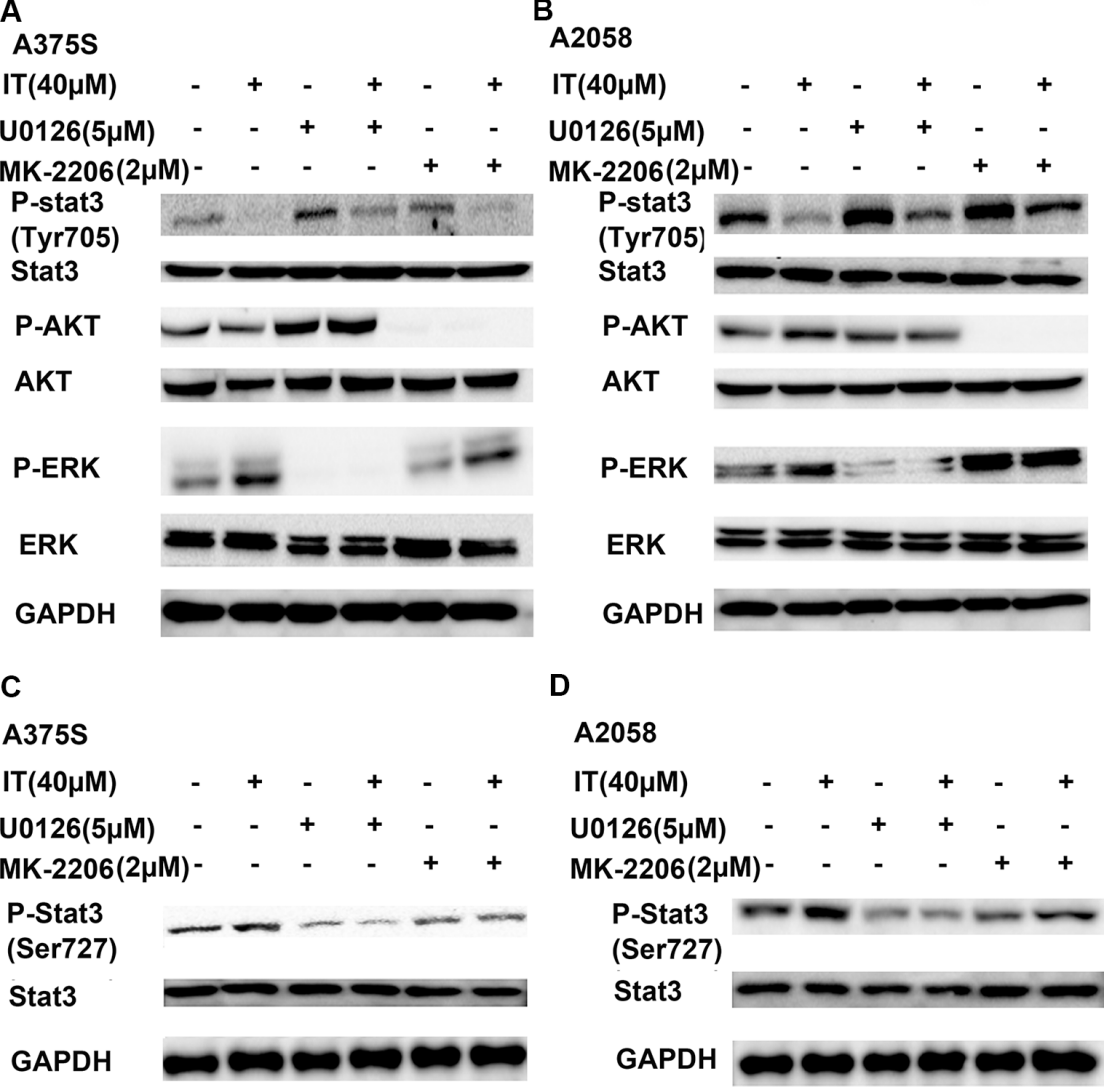
Blockade of AKT and ERK activation partially reversed IT-induced STAT3 inhibition A375S (**A**, **C**) and A2058 (**B**, **D**) cells were pretreated with or without MK-2206 (2 μM) or U0126 (5 μM) for 1 h, then the cells were treated with or without IT (40 μM) for another 1 h. The cell lysates were extracted for western blot analysis using antibodies specific to p-AKT (ser473), AKT, p-ERK (Thr202/Tyr204), ERK, p-STAT3 (tyr 705), p-STAT3 (ser727), and STAT3.

AKT and ERK activation has been proved to inhibit STAT-transcriptional activities [26]. As shown in Figure 7A and 7B, treatment of MK-2206, an AKT inhibitor, alone or U0126, a MEK inhibitor, alone for 1 h markedly increased STAT3 phosphorylation at tyr705 site in human melanoma A375S and A2058 cells. MK-2206 or U0126 pretreatment for 1 h partially reversed IT (40 μM)-induced down-regulation of STAT3 phosphorylation at tyr705 site. STAT3 transcriptional activation can be controlled by STAT3 phosphorylation at ser727 site through the MAPK or AKT/mTOR pathways [29–31]. We also observed that IT treatment for 1 h significantly increased the phosphorylated STAT3 (ser727) in human melanoma A375S and A2058 cells, while MK-2206 or U0126 pretreatment for 1 h partially reversed IT(40 μM)-inducedSTAT3 phosphorylation at ser727 site (Figure 7C, 7D).

### IT inhibited IGF-1- induced STAT3 activation in melanoma cells

**Figure 8:**
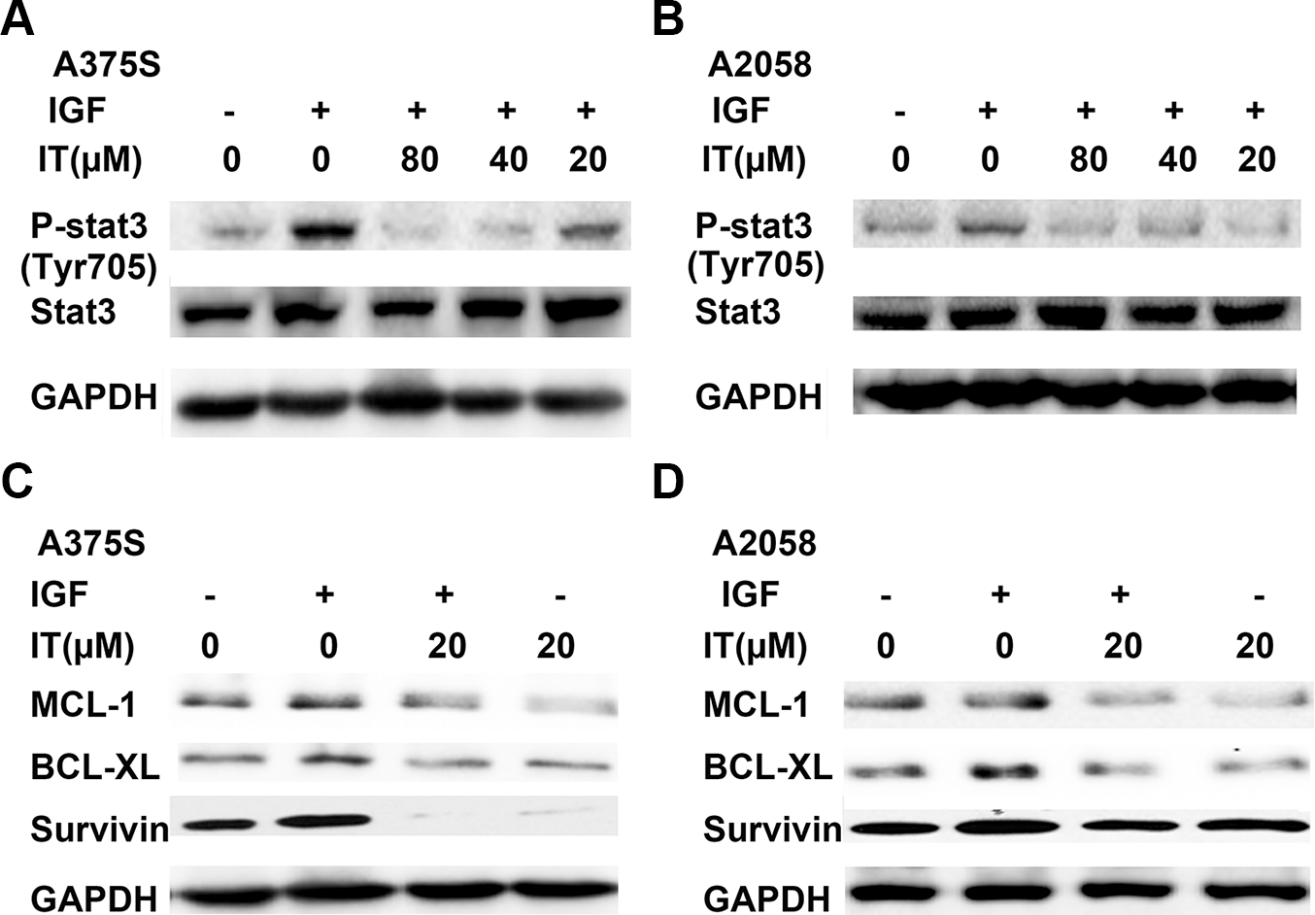
IT inhibited IGF-1- induced STAT3 activation and STAT3 target genes expression For p-STAT3 analysis, A375S (**A**) and A2058 (**B**) cells were grown in serum-free medium, and pretreated with IT (20, 40, and 80 μM) or vehicle control for 2 h, then the cells were stimulated with IGF-1 (20 ng/mL) for 1 h. The cell lysates were extracted for western blot analysis using antibodies specific to p-STAT3 (tyr705), and STAT3. For STAT3 target genes assay, A375S (**C**) and A2058 (**D**) cells were grown in serum-free medium, and pretreated with IT (20 μM) for 2 h, then the cells were stimulated with or without IGF-1 (20 ng/mL) for 24 h. The cell lysates were extracted for western blot analysis using antibodies specific to BCL-XL, MCL-1, and survivin.

It has been well known that STAT3 could be activated by IGF-1 stimulation. To determine if IT treatment inhibited IGF-1-induced STAT3 activation, melanoma cells were grown in serum-free medium for 24 h, and then pretreated with or without IT (20, 40 and 80 μM) for 2 h, followed by addition of IGF-1 (20 ng/mL) for 1 h. The cell lysates were extracted for western blot analysis. As shown in Figure 8A and 8B, IGF-1 stimulation for 1 h markedly increased STAT3 phosphorylation at tyr705 site, while IT pretreatment significantly inhibited IGF-1- induced STAT3 activation in A375S and A2058 cells.

### IT inhibited IGF-1- induced STAT3 target genes expression in melanoma cells

To examine the effects of IT on STAT3- targeted genes upon IGF-1 stimulation, melanoma cells were grown in serum-free medium for 24 h, and then pretreated with or without IT (20 μM) for 2 h, followed by stimulation with IGF-1 (20 ng/mL) for another 24 h. The cell lysates were then extracted for western blot analysis. As demonstrated in Figure 8C and 8D, Stimulation with IGF-1 for 24 h significantly increased the expression levels of STAT3-targeted genes, including survivin, BCL-XL, and MCL-1, while IT pretreatment for 2 h markedly decreased IGF-1-induced survivin, BCL-XL, and MCL-1 expression in human melanoma A375S and A2058 cells.

### IT reduced the expression levels of phospho-IGF-1R and total IGF-1R in melanoma cells

**Figure 9:**
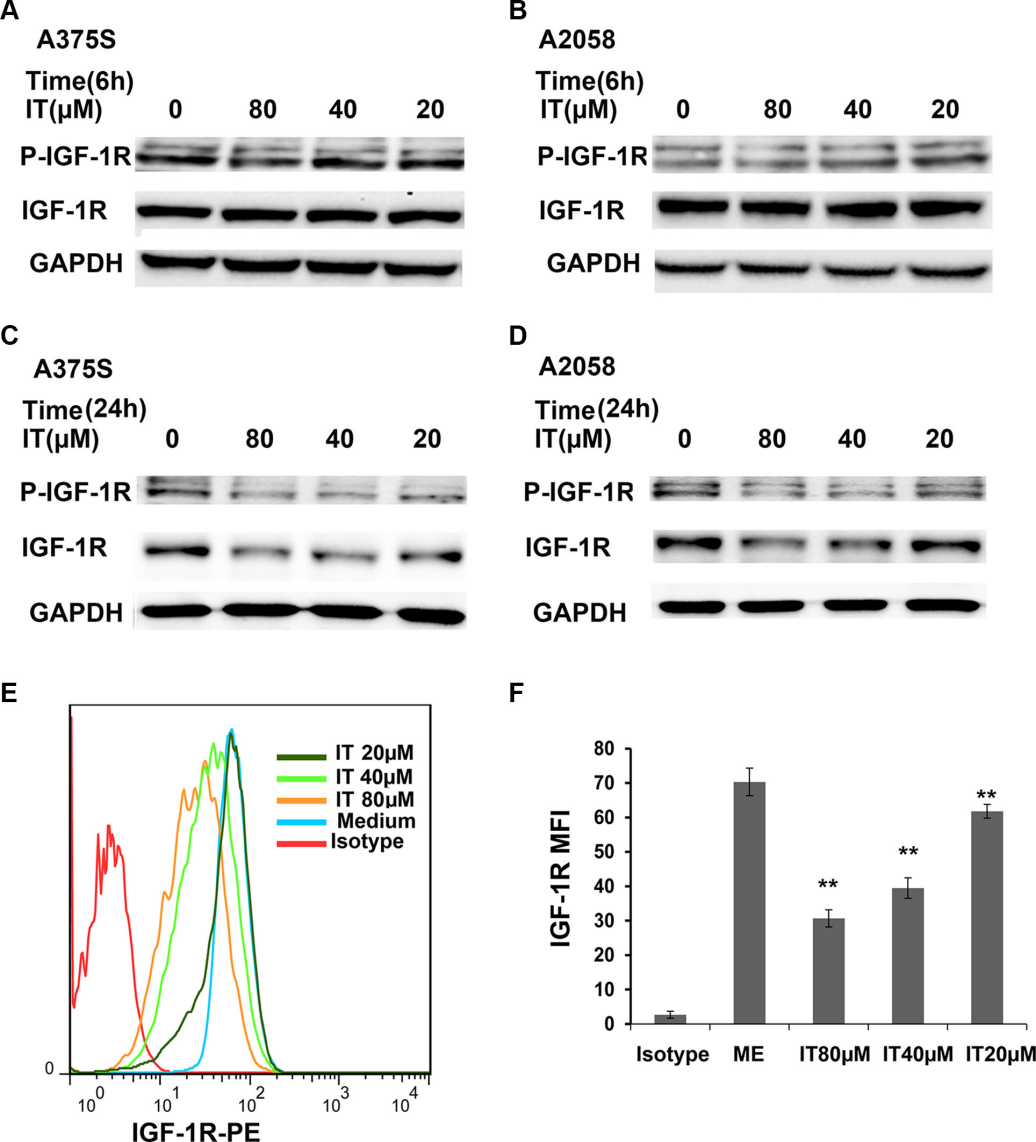
IT reduced the expression levels of phospho-IGF-1R and total IGF-1R in melanoma cells A375S cells and A2058 cells were treated with IT (20, 40, and 80 μM) or vehicle control for 6 h (**A**, and **B**) or 24 h (**C**, and **D**). The cell lysates were extracted for western blot analysis using antibodies specific to p-IGF-1R (Tyr1135/1136), and IGF-1R. A375S cells were treated with IT for 72 h, and then the cells were stained with PE conjugated IGF-1R antibody or PE conjugated isotype control antibody. The mean fluorescence intensity (MFI) data was analyzed by FlowJo software V6.0 (Tree star, Ashland, OR). (**E**) Representative images of IGF-1R MFI. (**F**) Statistical data of IGF-1R MFI. The MFI data were replicated three times. **indicates *P* < 0.01, as compared with isotype control group.

To further clarify the role of IT on IGF-1R signaling, the expression levels of phospho-IGF-1R and total IGF-1R in A375S and A2058 cells under IT treatment were determined by western blot. As shown in Figure 9A and 9B, IT treatment (20, 40 and 80 μM) for 6 h did not change the expression levels of phospho-IGF-1Rβ (Tyr1135/1136) and total IGF-1Rβ, while IT treatment for 24 h markedly reduced the expression levels of phospho-IGF-1Rβ (Tyr1135/1136) and total IGF-1Rβ (Figure 9C and 9D). Flow cyotmetry analysis was employed to further verify the effects of IT on IGF-1R expression. As shown in Figure 9E and 9F, IT treatment (20, 40 and 80 μM) reduced the expression of IGF-1R in a dose-dependent manner (*P* < 0.01).

### IT inhibited IGF-1-induced activation of IGF-1R through down-regulation of total IGF-1R in melanoma cells

**Figure 10:**
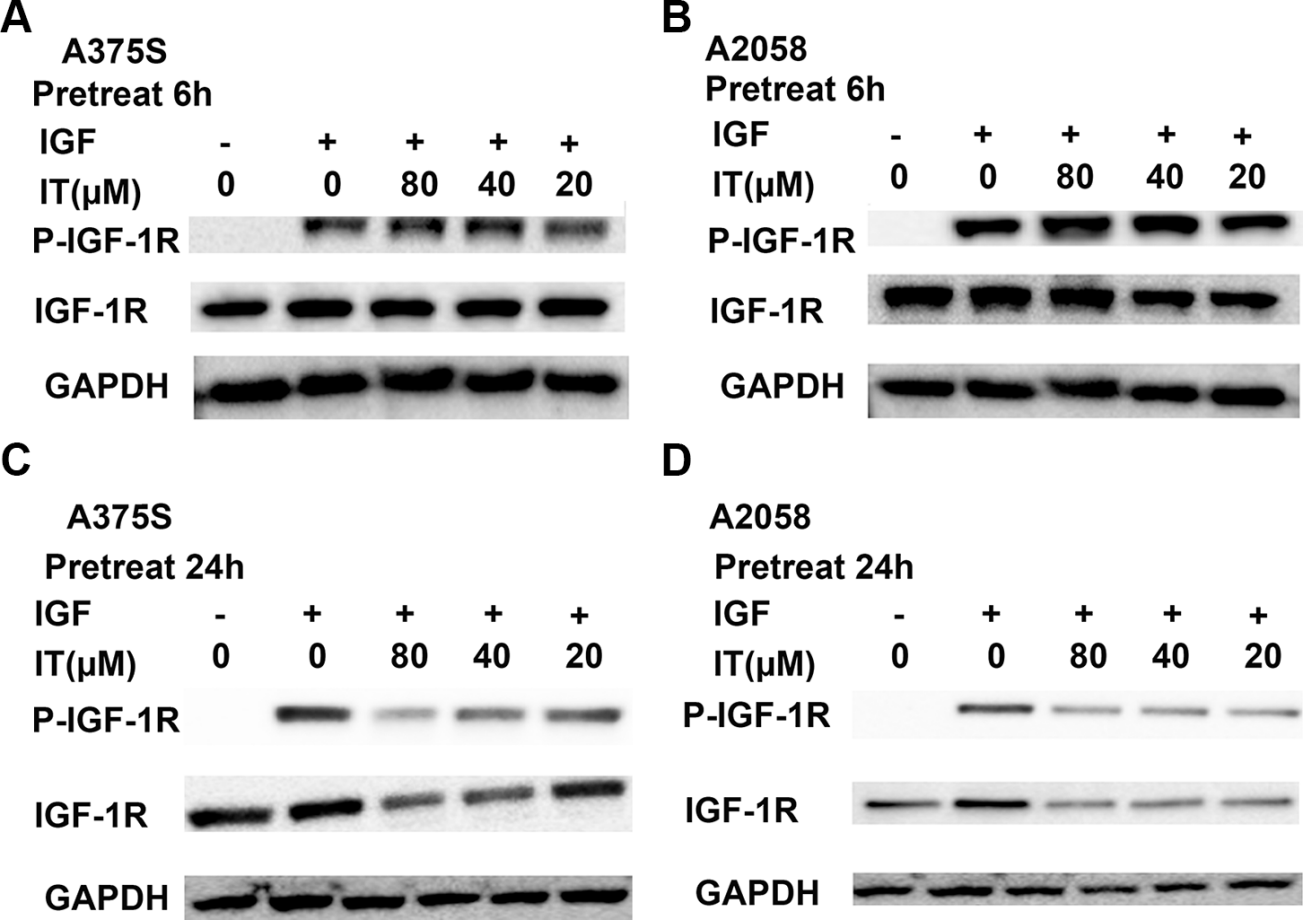
IT inhibited IGF-1-induced activation of IGF-1R most likely through decreases of total IGF-1R A375S and A2058 cells were grown in serum-free medium, and pretreated with IT (20, 40, and 80 μM) or vehicle control for 6 h (**A**, and **B**) or 24 h (**C**, and **D**), and then stimulated with IGF-1 (20 ng/mL) for 15 min. The cell lysates were extracted for western blot analysis using antibodies specific to p-IGF-1R, and IGF-1R.

To eliminate the effects of serum growth factors, A375S and A2058 melanoma cells were grown in serum-free medium for 24 h and then treated with either the vehicle or IT (20, 40 and 80 μM) for 6 h or 24 h before stimulating with IGF-1(20 ng/mL) for 15 min. As shown in Figure 10A and 10B, IT pretreatment for 6 h did not reduce the levels of phosphorylatedIGF-1R in the IGF-1-stimulated melanoma cells, while IT pretreatment for 24 h significantly decreased the phosphorylated IGF-1R in the IGF-1-stimulated melanoma cells (Figure 10C and 10D). In addition, total IGF-1R expression levels were markedly reduced by IT pretreatment for 24 h in the IGF-1-stimulated melanoma cells (Figure 10C and 10D).

### Overexpression of IGF-1R partially reversed IT-induced growth inhibition in melanoma cells

**Figure 11:**
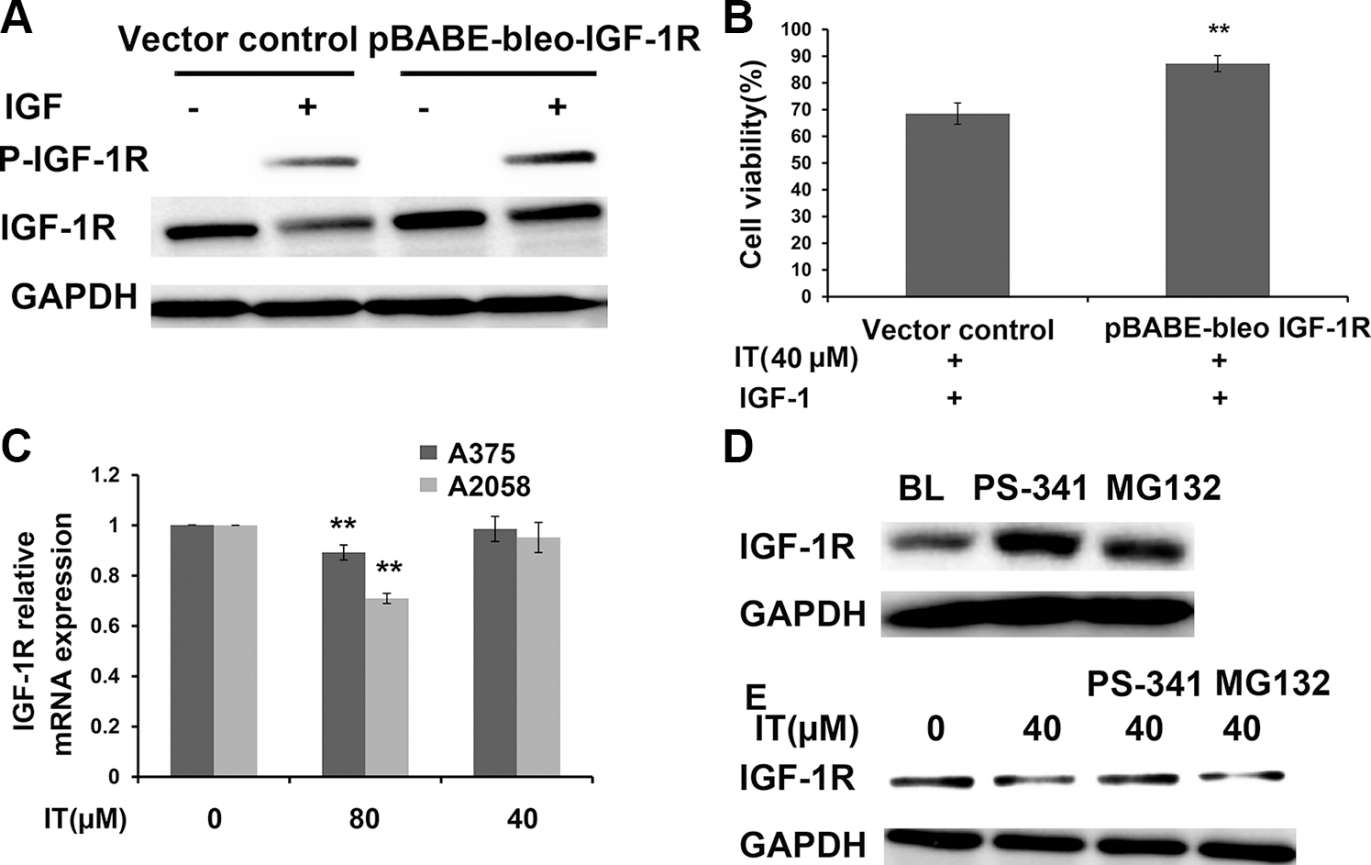
Overexpression of IGF-1R partially reversed IT-induced cell growth inhibition A375S cells were transiently transfected with pBABE-bleo IGF-1R or pcDNA for 48 h. (**A**) the transfected cells were stimulated with or without IGF-1(100 ng/mL) for 15 min. The cell lysates were extracted for western blot analysis using antibodies specific to p-IGF-1R, and IGF-1R. (**B**) The transfected cells were treated with IT(40 μM) and stimulated with IGF-1 (100 ng/mL) for 24 h, and then cell viability was determined by MTT assay. **indicates *P* < 0.01, as compared with vector control. (**C**) IT slightly inhibited IGF-1R transcription. A375s and A2058 cells were treated with IT (40, 80 μM) or vehicle control for 24 h. The total RNA was extracted, and then real-time PCR analysis was performed to detect the expression of IGF-1R mRNA. **indicates *P* < 0.01, as compared with vehicle control. (**D**) Proteasome inhibitors blockaded IGF-1R degradation. A375S cells were treated with PS-34 1 (20 nM) or MG132 (10 μM) alone for 6 h. The cell lysates were extracted for western blot analysis using antibody specific to IGF-1R. (**E**) Proteosomal degradation pathway was involved in IT-induced loss of IGF-1R. A375S cells were pretreated with IT (40 μM) for 16 h, and then PS-341 (20 nM) or MG132 (10 μM) was added for another 6 h. The cell lysates were extracted for western blot analysis using antibody specific to IGF-1R.

To further clarify whether IT-induced melanoma growth inhibition is correlated with down-regulation of IGF-1R, A375 cells were transiently transfected for 48 h with pBABE-bleo IGF-1R or vector control. Western blot (Figure 11A) analysis demonstrated that transfection of pBABE-bleo IGF-1R in A375 cells resulted in a remarkable increase in IGF-1R level, as compared with that in vector control group. The phosphorylation level of IGF-1R in pBABE-bleo IGF-1R transfection group was significantly elevated after IGF-1 stimulation, as compared with that in vector control group. After IT treatment and IGF-1 stimulation for 24 h, the cell viability in pBABE-bleo IGF-1R group was partially rescued, as compared with that in vector control group (Figure 11B, *P* < 0.01).

### IT slightly inhibited IGF-1R transcription in melanoma cells

To determine if IT would inhibit IGF-1R at transcriptional level, we performed real-time PCR assay to detected IGF-1R mRNA expression from A375S and A2058 cells cultured alone or in the presence of IT (40, 80 μM) for 24 h. The real-time PCR data indicated that IT 80 μM treatment slightly reduced IGF-1R mRNA levels in A375S and A2058 cells, while IT 40 μM treatment did not alter the levels of IGF-1R mRNA in A375S and A2058 cells (Figure 11C). The modest decreases of IGF-1R mRNA (10% or 25% decreases in IT 80 μM treated A375 or A2058 cells, respectively) compared with the dramatic loss of IGF-1R protein (50% or 55% decreases in IT 80 μM treated A375 or A2058 cells, respectively), suggested that IT acted primarily at post-transcriptional level.

### Proteosomal degradation pathway was involved in IT-induced loss of IGF-1R in melanoma cells

Proteosomal degradation pathway is one of the major pathways involved in protein degradation. To determine the involvement of proteosomal degradation pathway in IT-induced IGF-1R protein down-regulation, we pretreated melanoma cells with MG132, an inhibitor of the 26S proteosome, and PS-341, an inhibitor of the 20S proteosome, and detected whether IT-induced IGF-1R down-regulation could be rescued. As shown in Figure 11D, MG132 or PS-341 treatment alone for 6 h significantly increased the expression level of IGF-1R, while only PS-341 treatment for 6 h rescued IT-induced IGF-1R down-regulation (Figure 11E).

### Inhibition of FASN suppressed IGF-1R expression in melanoma cells

**Figure 12:**
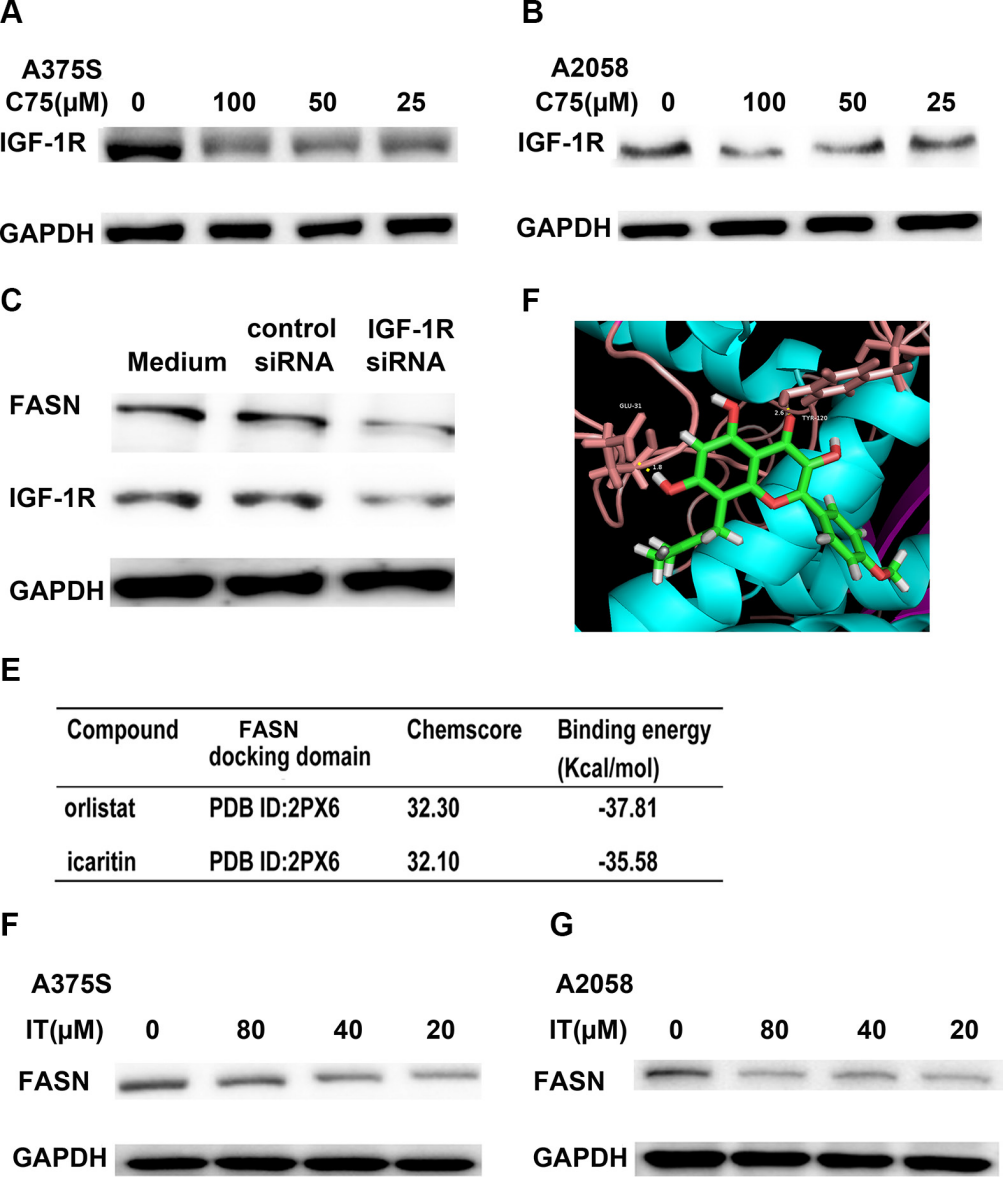
IT-induced IGF-1R loss was partially through FASN inhibition A375S (**A**) and A2058 cells (**B**) were treated with different concentration of C75 (25, 50, and 100 μM) or vehicle control for 24 h. The cell lysates were extracted for western blot analysis by using antibody specific to IGF-1R. (**C**) FASN siRNA transfection decreased IGF-1R expression in melanoma cells. A375S cells were transiently transfected with FASN siRNA or scrambled siRNA for 48 h. The cell lysates were extracted for western blot analysis using antibodies specific to FASN and IGF-1R. (**D**) The optimal binding conformation of the FASN- icaritin complexes. (**E**) The chemscore and binding energy of each complex. The docking studies of FASN with icaritin or reference ligand (orlistat) were performed using the same GOLD parameters. A375S (**F**) and A2058 cells (**G**) were treated with different concentration of IT (20, 40, and 80 μM) or vehicle control for 24 h. The cell lysates were extracted for western blot analysis using antibody specific to FASN.

Inhibition of fatty acid synthase (FASN) has been reported to decrease the expression of c-Met, a tyrosine kinase receptor [32]. Therefore, we examined if inhibition of FASN could suppress IGF-1R expression in melanoma. As shown in Figure 12A and 12B, treatment of C75 (25, 50 and 100 μM), a FASN inhibitor, for 24 h markedly decreased the levels of IGF-1R. To further determine the role of FASN inhibition in IGF-1R expression, we used FASN siRNA transfection to knockdown FASN and detected the IGF-1R expression by Western Blot analysis. As demonstrated in Figure 12C, a remarkable decrease of IGF-1 R was observed in FASN siRNA transfected cells, as compared with control siRNA transfected cells.

### IT is a novel FASN inhibitor

To determine whether IT is a potential FASN inhibitor, we performed homology modeling by using GOLD suite v5.3 to obtain information regarding binding energies. Orlistat, a FASN inhibitor, was selected as reference ligand. The docking studies of FASN with icaritin or reference ligand were performed using the same GOLD parameters. The optimal binding conformation of the FASN-icaritin complexes were presented in Figure 12D, as well as the chemscore, and binding energy of each complex were shown in Figure 12E. Molecular docking analysis showed that IT had similar chemscore and binding energy to orlistatat in FASN docking domain (PDB ID 2PX6). Western blot data demonstrated that IT markedly suppressed the expression of FASN in A375S and A2058 melanoma cells (Figure 12F, 12G).

### Palmitate partially rescued IT-induced IGF-1R decreases and growth inhibition in melanoma cells

**Figure 13:**
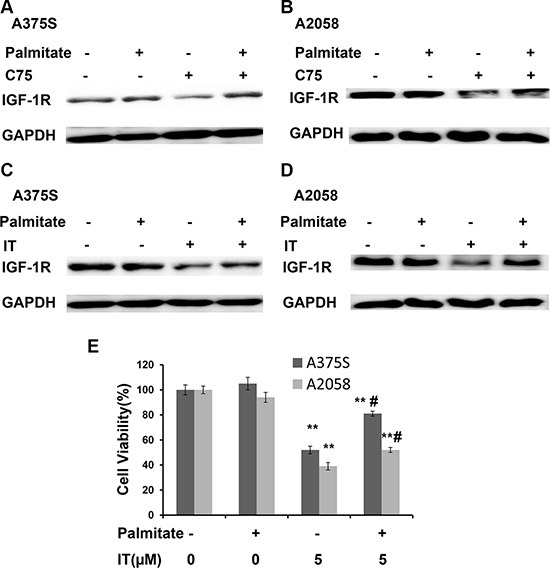
Palmitate partially rescued IT-induced IGF-1R down-regulation A375S (**A**) and A2058 (**B**) cells were treated with C75 (50 μM) or palmitate (100 μM) or combination of C75 and palmitate for 24 h. A375S (**C**) and A2058 (**D**) cells were treated with IT (40 μM) or palmitate (100 μM) or combination of IT and palmitate for 24 h. The cell lysates were extracted for western blot analysis using antibody specific to IGF-1R. (**E**) A375 and A2058 cells were grown in serum-free medium, and treated with IT (5 μM) or palmitate (100 μM) or combination of IT and palmitate for 48 h. The cell viability was determined by MTT assay. **indicates *P* < 0.01, as compared with vehicle control. # indicates *P* < 0.01, as compared with IT treatment alone group.

Palmitate is one of terminal products of FASN. Western blot (Figure 13A–13D) showed that addition of 100μM palmitate partially rescued C75 (50 μM) or IT (40 μM)-induced IGF-1R decreases in A375S and A2058 melanoma cells, respectively. As demonstrated in Figure 13E, palmitate treatment partially rescued IT (5 μM)-induced growth inhibition in A375S and A2058 melanoma cells (*P* < 0.01).

## DISCUSSION

Chinese herb has been increasingly used in the last decades and become well known for its significant role in preventing and treating cancer [33]. In this study, we firstly examined the anti-melanoma activities of IT, a flavonoid isolated from *Herba Epimedii*. MTT data demonstrated that IT exerted cytotoxicity to melanoma cells in a time- and dose-dependent manner, while IT treatment only showed minor cytotoxicity to human normal skin fibroblast cells. These data suggested that IT might be a novel anti-melanoma agent with low cytotoxicity to normal cells.

STAT3 activation is involved in regulating cell proliferation, angiogenesis, metastasis and inhibition of apoptosis in melanoma [32]. We found that IT significantly inhibited STAT3 activity in melanoma cells. Accordingly, the levels of STAT3-targeted genes, such as survivin, Mcl-1, and Bcl-xL, were down-regulated after IT treatment. Overexpression of STAT3 partially reversed IT-induced melanoma growth inhibition, which further suggested that STAT3 signaling was involved in anti-melanoma activities of IT.

STAT3 can be activated by growth factors (EGF, IGF-1, and PDGF, etc) [12]. The biological actions of IGF-1 are mediated through the ligand-induced activation of IGF-1R. We found that IT treatment for 24 h decreased both p-IGFR and IGF-1R expression levels. To eliminate the influence of serum growth factors, melanoma cells were grown in serum-free medium and then treated with IT before stimulating with IGF-1. Interestingly, the reduction of IGF-1-induced phosphorylation of IGF-1R appeared to be due to IT-induced down-regulation of total IGF-1R protein. Overexpression of IGF-1R partially reversed IT-induced cell growth inhibition, which suggested that IGF-1R signaling was involved in the anti-melanoma activities of IT.

Real-time PCR analysis showed that no dramatic changes in IGF-1R mRNA levels were observed after IT treatment, suggesting that down-regulation of IFG-1R expression by IT at post-transcriptional level. Proteasome degradation pathway is one of the major pathways involved in protein degradation [34, 35]. Using PS-341, a specific 20S proteasome inhibitor, we found that IT-induced IGF-1R protein reduction was partially reversed. These data suggested that proteasome degradation pathway might be involved in IT-induced IGF-1R degradation.

FASN is the sole enzyme responsible for *de novo* synthesis of long-chain unsaturated fatty acids, primarily the 16-carbon fatty acid palmitate [36]. Many human cancers exhibit increased FASN expression [36]. Using molecular docking analysis, IT was identified as a novel FASN inhibitor. Western blot analysis showed that IT markedly inhibited FASN expression in melanoma cells. C75, a specific pharmacologic inhibitor to FASN, as well as FASN-specific siRNA transfection reduced the level of total IGF-1R, which suggested that IT- induced IGF-1R reduction was through FASN inhibition. The addition of exogenous palmitate, a terminal product of FSAN, to the system partially rescued the IT-induced down-regulation of IGF-1R, further supporting the role of FASN in maintaining IGF-1R expression levels.

However, the mechanism of how FASN inhibition decreases IGF-1R expression is not yet clear. Several cellular receptors, including IGF-1R, require localization within lipid rafts for efficient signaling [37]. Lipid rafts are rich in cholesterol and sphingolipids, products generated in tumors cells by FASN [38]. IT induced-FASN inhibition may cause an imbalance in the membrane lipids levels, which may result in decreased IGF-1R membrane localization and inactivation of the downstream STAT3 signaling.

PI3K/AKT/mTOR and Ras/Raf/MEK/ERK are two major downstream cascades of IGF-1R signaling [39]. These two pathways are activated in melanoma and promote melanoma development [28]. Unexpectedly, IT treatment activated AKT and ERK signaling in melanoma cells. The effects of IT on AKT and ERK signaling in caners are controversial. Zhu et al and Li et al observed that IT inhibited the activation of ERK and AKT in human leukemia cells [25, 40].Tong et al and Guo et al reported that IT induced sustained phosphorylation of ERK in human endometrial cancer cells and human breast cancer cells [22, 41]. Wu et al revealed that IT activated AKT in human lung cancer cells [42]. Different roles of IT on AKT and ERK signaling might be cell type dependent.

It has been reported that ERK and AKT signaling inhibited STAT3 activities in human melanoma cells [26]. Our data showed that AKT inhibitor or ERK inhibitor treatment alone markedly increased STAT3 phosphorylation at tyr705 site. We also found that AKT or ERK blockade partially reversed IT-induced decrease of STAT3 phosphorylation (tyr705). These data suggested that IT-induced STAT3 inhibition partially through activation of AKT and ERK signaling in melanoma cells. The mechanism of IT-induced activation of AKT and ERK signaling need to be further investigated.

In summary, we reported that IT exerted anti-melanoma activities. These effects were, at least partially, due to the inhibition of FASN/IGF-1R/STAT3 signaling. Our findings provided novel insights into the anti-melanoma mechanisms of IT, and further suggested a potential role of IT in melanoma management.

## MATERIALS AND METHODS

### Reagents

Icaritin (IT, > 99% pure) was purchased from Shanghai Ronghe Co (Shanghai, China). Vemurafenib (PLX4032) was obtained from LC laboratory (Woburn, MA). MTT [3-(4,5-Dimethylthiazol-2-yl)-2,5-Diphenyltetrazolium Bromide],and crystal violet were supplied by Sigma-Aldrich (St. Louis, MO). The antibodies against PARP, survivin, Bcl-XL, Mcl-1, phosphorylated (P)-STAT3(tyr705, and ser727), STAT3, P-ERK (Thr202/Tyr204), ERK, P-AKT (ser 473), AKT, P-IGF-1Rβ (Tyr1135/1136), IGFRβ, Fatty acid synthase (FASN), PCNA and GAPDH were supplied by Cell Signaling Technology (Beverly, MA). MK-2206 and U0126 were obtained from SelleckChem (Houston, TX). Human recombinants IGF-1 was supplied by PeproTech (Rocky Hill, NJ). Annexin V-fluorescein isothiocyanate (FITC)/propidiumiodide (PI) apoptosis detection kit was purchased from BD Biosciences (San Jose, CA).

### Cell culture

The human melanoma A375, A2058, MEWO and human skin fibroblast HS68cells were purchased from American Type Culture Collection (Manassas, VA) and maintained in DMEM (Invitrogen, Carlsbad, CA) containing 4 mML-glutamine, 3.7 g/L sodium bicarbonate, 4.5 g/L glucose and 5% fetal bovine serum (FBS, Invitrogen, Carlsbad, CA). Cells were maintained in a 5% CO2 humidified incubator at 37°C. To generate melanoma cell line with BRAF inhibitor acquired resistance, A375 parental cells (A375S), exquisitely sensitive to vemurafenib, were chronically exposed to incremental increases of vemurafenib for 6 months until a subline grew progressively as described [43, 44]. Vemurafenib-resistant cells (A375R) were cloned in 5 μM vemurafenib, a concentration at which parental cells were not viable. For culturing cells in the presence of palmitate-bovine serum albumin (BSA) complex, palmitate (Sigma, St. Louis, MO) was first complexed to fatty acid–free BSA (Sigma, St. Louis, MO) as described [45, 46]. Briefly, 4 volumes of 4% BSA solution in 0.9% NaCl were added to 1 volume of 5 M palmitate in ethanol and incubated at 37°C for 1 h, to obtain a 1 M stock solution of BSA-complexed palmitate.

### Cell viability assays

The cytotoxic effects of IT on A375S, A375R, A2058, MEWO and HS68 cells were determined by MTT assay. Cells (4000/200 μL/well) were seeded in 96-well plates, and treated with either vehicle control (DMSO) or various concentrations of IT (2.5, 5, 10, 20, 40, and 80 μM). After incubation for 24, 48 or 72 h, 20 μL of MTT solution (5 mg/mL) was added to each well and incubated for 2 h. The formazan crystal formed was dissolved with 100 μL of DMSO; absorbance was detected at 570 nm by a microplate spectrophotometer (BD Biosciences, San Jose, CA). For Crystal Violet assay, cells (2.5 ×10^5^) were seeded in 60 mm dishes, and exposed to IT (20, 40, and 80 μM) or vehicle control for 72 h, then cells were fixed with 10% formalin for 10 min, followed by staining with 0.05% crystal violet solution (CV) in distilled water for 30 min. Finally, CV was removed, the cells were washed twice with distilled water and images were photographed.

### Apoptosis assay

Apoptosis in melanoma cells were evaluated by Annexin V/PI double staining with the Apoptosis Detection Kit, according to the manufacturer's instructions. Briefly, cells were harvested after IT (0, 20, 40, 80 μM) treatment for 24 h and 1 × 10^5^ cells were then incubated in 100 μL labeling solution (5 μL of Annexin V-FITC, 5 μL of PI, 10 μL of 10 × binding buffer and 80 μL of H_2_O) in darkness at room temperature for 15 min, after that, 400 μL of 1 × binding buffer was added to stop the staining reaction. Flow cytometric analyses were performed on a FACS Calibur^™^ (BD Biosciences, San Joe, CA) utilizing 10,000 events. The data was analyzed by FlowJo software V6.0 (Tree star, Ashland, OR).

### Western blot analysis

For total protein extractions, cells were harvested and resuspended in lysis buffer (150 mmol/LNaCL, 1% NP-40, 0.5% sodium deoxycholate, 0.1% SDS, and50 mmol/L Tris-Cl pH 8.0, 2 ug/mL aprotinin, 2 ug/mL leupeptin, 40 mg/mL of phenylmethylsulfonyl fluoride, 2 mmol/LDTT) and centrifuged at 12,000 × g for 15 min. Supernatants were then quickly frozen at −80°C until use. Nuclear proteins were extracted, according to our previously reported procedures [47]. The protein concentration was determined by the Bradford assay (Biorad, Hercules, CA). 30 μg ofcellular proteins were electroblotted onto a PVDF membrane following separation on a 10% SDS-polyacrylamide gel electrophoresis. The immunoblot was incubated 1 h with 5% non-fat milk at room temperature, followed by an overnight incubation at 4°C with a 1:1000 dilution of corresponding primary antibodies. Blots were washed twice with Tris buffered saline/Tween 20 (TBST) before addition of a 1:3000 dilution of HRP-conjugated secondary antibody for 1 h at room temperature. Blots were again washed with TBST before development by enhanced chemiluminescence using Supersignal West Femto Chemiluminescent Substrate (Pierce, Rockford, IL).

### Immunofluorescent staining analysis

Immunocytofluorescent staining analysis was performed to assess the expression of STAT3 at the protein level and its localization. Briefly, A375 cells cultured in 6-well plates were treated with IT (0, 20, and 40 μM) for 6 h, and then the cells were fixed with 4% paraformaldehyde for 15 minutes at room temperature, washed with PBS/5% Tween, and permeabilized with cold methanol. After washing, the cells were blocked in 10% goat serum/PBS, and stained with monoclonal rabbit anti-STAT3 (1:1600) at 4°C overnight. Cells were stained with a secondary Alexa Fluor-488 labeled goat anti-rabbit IgG (1:500, abcam, Cambridge, UK) and counterstained with 1 μg/ml 4,6-diamino2-phenyl indole dihydrochloride (DAPI, sigma, St. Louis, MO). Cells were visualized using an Olympus fluorescent microscope.

### Plasmid transient transfection

The constitutive activated STAT3 expression constructs (Stat3-C), pBABE-bleo IGF-1R, and vector (pcDNA) plasmids were obtained from Addgene(Cambridge, MA). Transfection of plasmids into melanoma cells were conducted by using Lipofectamine 2000 (Invitrogen, Carlsbad, CA) following the manufacturer's protocol. Cells were transfected with plasmids for 48 h before functional assays were carried out.

### Transient transfection with siRNA

SignalSilence^®^ FASN siRNA (CST, Beverly, MA) was used to knock down FASN. SignalSilence^®^ Control siRNA (Unconjugated, CST) was used as a negative control. Transfection of siRNA into melanoma cells was conducted by using Lipofectamine 2000 (Invitrogen, Carlsbad, CA) following manufacturer's protocol. Cells were transfected with siRNA for 48 h before functional assays were carried out.

### Real-time PCR assay

Total RNA was extracted with Trizol reagent (Invitrogen, Carlsbad, CA), and reverse transcription was performed to obtain the cDNA using the Prime Script RT reagent Kit (Takara, Japan), according to the manufacturer's protocol. The primers used were synthesized by Invitrogen (Carlsbad, CA). These quences were as follows: Human IGF-1Rβ: 5′-CTCCTGTTTCTCTCCGCCG-3′ (forward) and 5′-ATA GTCGTTGCGGATGTCGAT-3′ (reverse);GAPDH: 5′-CT GCACCACCAACTGCTTAGC-3′(forward)and 5′-CTTC ACCACCTTCTTGATGTC-3′(reverse). Quantitative real-time PCR was performed using SYBR green reaction mixture in theViiA^™^7 Real Time PCR System (Applied Biosystems, Grand Island, NY). The relative expression levels were calculated using 2^−ΔΔCt^ methods.

### Flow cytometry analysis

After treatment with different concentrations of IT for 72 h, the cells were harvested and transferred to flow tubes. Cells were washed and resuspended in staining buffer (0.5%BSA, diluted in PBS). Cells were then stained with PE conjugated anti-IGF-1R monoclonal antibody (# 555999, BD Biosciences, San Jose, CA) or PE conjugated isotype control (#555749) in the dark for 30 min on ice. After washing twice, the cells were resuspended in 300 μL of staining buffer. Data was acquired on a FACS Calibur TM (BD Biosciences, San Joe, CA) utilizing 20,000 events. The mean fluorescence intensity (MFI) data was analyzed by FlowJo software V6.0 (Tree star, Ashland, OR).

### Molecular docking analysis

Molecular docking can fit molecules together in a favorable configuration to form a complex system. Structural information from a theoretically modeled complex may help us clarify the binding mechanism between FASN and icaritin (CID: 5318980). FASN-inhibitor complexed X-ray crystallographic structures (PDB ID: 2P × 6) was selected as a starting structure for the prediction of binding site of FASN. Orlistat, the original ligand in FASN-inhibitor complex, was used as a reference ligand for verifying the binding ability of icaritin. Molecular docking was performed by GOLD suite v5.3. To investigate the full range conformational flexibility of ligand with partial flexibility of the receptor, GOLD uses genetic algorithm for docking ligand into binding site of target protein. The ligand binding energy with target protein was predicted via chemscore and free energy of binding implemented in GOLD.

### Statistical analysis

All data were presented as the mean ± standard deviation. Data analysis was performed by one-way analysis of variance *(ANOVA)*. For comparison of two groups, a student's *t*-test was used. Differences with *P* values < 0.05 were considered to be statistically significant.
